# Remote intestinal changes following blast-induced thoracic trauma with transient hemorrhagic shock and hind-limb ischemia

**DOI:** 10.1186/s40635-026-00970-w

**Published:** 2026-09-18

**Authors:** Sophie Meisen, Lena Doerfer, Marco Mannes, Annette Palmer, Frank Hildebrand, Alexander Kleger, Rebecca Halbgebauer, Markus Huber-Lang

**Affiliations:** 1 Institute of Clinical and Experimental Trauma-Immunology, Ulm University Medical Center, Helmholtzstr. 8/1, 89081 Ulm, Germany; 2 Department of Orthopedics, Trauma and Reconstructive Surgery, RWTH Aachen University Hospital, Aachen, Germany; 3 Institute of Molecular Oncology and Stem Cell Biology, Ulm University Hospital, Ulm, Germany

**Keywords:** Remote trauma, Intestinal injury, Gut-blood-barrier dysfunction

## Abstract

**Background:**

The intestine is both a driver and a target of posttraumatic organ dysfunction. However, it remains unclear whether, and to what extent an extra-abdominal trauma, such as thoracic trauma with transient hemorrhagic shock and hind-limb ischemia (THS), provokes remote intestinal alterations.

**Methods:**

A post hoc analysis was performed using jejunal tissue from a standardized murine THS model. Histological, immunohistochemical, and molecular analyses evaluated epithelial integrity, barrier components, and immune responses 24 h after the insult.

**Results:**

Histological assessment of the intestine revealed no overt morphological damage. Nonetheless, jejunal expression of *Tnk1* and *Ripk3* was significantly increased, indicating epithelial stress and activation of necroptotic signaling. Tight junction marker *Tjp1* was upregulated, while *Muc2* was reduced. Intestinal expression levels of *Il6* and *Ifng* declined, paralleled by a decrease in CD3⁺ T cells, whereas *Lcn2* levels increased.

**Conclusions:**

Thoracic trauma combined with transient hemorrhagic shock including hind-limb ischemia induces remote intestinal alterations, characterized by epithelial stress, compensatory barrier reinforcement, and reduced mucosal immunity. These findings highlight the gut’s sensitivity to extra-abdominal trauma and its potential contribution to systemic injury propagation.

**Supplementary Information:**

The online version contains supplementary material available at https://doi.org/10.1186/s40635-026-00970-w.

## Introduction

Direct intestinal injury, such as ischemia-reperfusion, can trigger remote organ dysfunction, including acute lung injury [1, 2]. Conversely, the intestinal tract can itself become a remote target following extra-abdominal trauma. Mechanistically, the inflicting traumatic force vector associated with severe tissue injury disrupts both macro- and micro-barriers, leading to the release of damage-associated molecular patterns (DAMPs), exposure to microorganisms, and their microbe-associated molecular patterns (MAMPs) [3]. The ensuing immune activation can initiate and perpetuate a vicious cycle of tissue destruction, immune dysregulation, thromboinflammation, barrier breakdown, edema, hypoxia, infection, and ultimately, multiple organ dysfunction syndrome (MODS) [4].

In the presence of concomitant hemorrhagic shock (HS), hemodynamic centralization compromises splanchnic perfusion, thereby driving intestinal dysfunction [5]. This condition induces Toll-like receptor 4 (TLR4) activation in intestinal epithelial cells, promoting high-mobility group box 1 (HMGB1) release and propagating remote organ failure [6, 7]. These processes contribute to the development of MODS [8].

Beyond its role in nutrient absorption, the intestinal tract harbors a vast and metabolically active microbial ecosystem [9]. Enterocytes, together with their associated tight junctions and mucus layers, form an “inner wall” separating the gut microbiome from the internal milieu, while mediating nutrient exchange and bidirectional communication with the host’s immune and nervous system [9, 10]. Upon severe trauma, direct or indirect damage to the gut-blood barrier (GBB) may result in the still-debated translocation of microbes, MAMPs, and DAMPs - either directly through the interstitium into the circulation or indirectly via intestinal lymphatic pathways [11]. According to the “gut-lymph-MODS” hypothesis, trauma-induced “toxic lymph” primes immune cells and thereby contributes to remote organ damage [12–14]. In line with this concept, rodents subjected to traumatic brain injury (TBI) developed a disrupted intestinal barrier and secondary endotoxemia [15]. Nevertheless, the precise mechanisms underlying intestinal and particularly GBB alterations following extra-abdominal trauma remain incompletely understood [16–18].

In a previous mouse study using a hemodynamically unstable severe polytrauma model, we observed intestinal dilatation, accompanied by increased GBB permeability and decreased expression of zonula occludens 1 (ZO-1) in the intestinal epithelium [19]. However, it remains unclear whether, and to what extent, a thorax trauma, one of the most common trauma entities, in the presence of transient HS and hind-limb ischemia results in remote intestinal morphological and functional alterations.

Therefore, we employed a standardized murine blast-induced trauma model, combined with transient HS and hind-limb ischemia, as recently published [20], and conducted a post hoc analysis in adherence to the 3R principles to evaluate potential remote intestinal structural and functional changes.

## Materials and methods

### Animals

A post hoc analysis was conducted on tissue samples obtained from a recently published study [20]. In brief, experimental blast-induced thoracic trauma combined with pressure-controlled hemorrhagic shock including hind-limb ischemia was performed on male C57BL/6J mice (Janvier Labs, Le Genest-Saint-Isle, France), aged 10 to 14 weeks and weighing 25.4 ± 0.3 g. Alongside with the experimental group (*n* = 8), a control group (*n* = 8) was included. All animals had ad libitum access to water, food, and nesting material. Housing conditions were maintained under a 14/10-hour light/dark cycle. All procedures involving animals were conducted in accordance with applicable animal welfare regulations and were approved by the Regional Council of Tübingen (Reg.-Nr. 1409).

### Anesthesia and application of blast-induced thoracic trauma followed by hemorrhagic shock

Mice received a subcutaneous bolus injection of buprenorphine (Temgesic^®^, Essex Pharma GmbH; 0.05 mg/kg body weight) 30 min prior to the induction of the experimental blast-induced thoracic trauma and the same dose of buprenorphine subsequently every 8 h throughout the whole observation period of 24 h post injury. The animals were set under anesthesia using a gas mixture of 3% sevoflurane (Abbott, Wiesbaden, Germany) and 97% oxygen and then placed in a dorsal position with limbs secured. For anatomical landmark orientation, the abdominal, thoracic and left femoral regions were shaved. Blast-induced thoracic trauma was applied during the inspiratory phase by a pressure wave, generated through the sudden release of compressed air for the duration of 1–2 ms from a cylinder positioned 1.6 cm above the murine sternum. The cylinder contained a polyester membrane (DuPont, Wilmington, USA) that ruptured upon a pressure of 0.83 ± 0.27 bar, supplied by a connected compressed air source. This led to the release of a focused pressure wave directed at the thoracic region, causing a blast-induced trauma without external injury. Subsequently, the mice were transferred to a heated platform, and rectal temperature was monitored and used for the feedback loop to maintain the core temperature at 37 °C. To induce pressure-controlled HS, the left femoral vessels were surgically exposed for the length of approximately 0.5 cm under aseptic conditions using a Leica surgical microscope (Leica Microsystems GmbH, Wetzlar, Germany). For local analgesia and vessel dilatation, one to two drops of lidocaine were applied to the femoral artery, after being ligated using 5 − 0 Silkam suture (Braun Surgical, Rubí, Spain). Cannulation of the femoral artery was performed using a curved 30G needle (Braun Melsungen, Melsungen, Germany) followed by insertion of a polyethylene catheter (ZUA-82; outer diameter: 0.62 mm, inner diameter: 0.28 mm; Föhr Medical Instruments GmbH, Seeheim/Ober-Beerbach) through the incision. The catheter was advanced into the femoral artery, allowing for administration of Jonosteril solution, a heparinized saline solution and measurement of the mean arterial pressure (MAP) and the heart rate. Vital parameters were recorded at 5 min-intervals throughout the procedure. Thirty minutes after induction of the thoracic trauma, HS was initiated by aspirating arterial blood into a 1 ml heparinized syringe. Blood withdrawal was continued for 5–10 min until a target MAP of 30 ± 5 mmHg was achieved and subsequently maintained at this level. A total of 829 ± 35 µl blood (mean ± SEM) per animal was withdrawn during the whole shock phase. This reflects a calculated ratio of 31.3 ± 0.8 µl/g body weight for the THS mice. After 60 min, heparinized withdrawn blood was transfused via the catheter and each mouse received two doses of 500 µl Jonosteril. Although listed in the protocol as resuscitation option, none of the animals required norepinephrine or other vasoactive drugs. Following catheter removal, the femoral artery was ligated, resulting in an additional hind-limb ischemia impact, and the surgical wound closed. Upon completion of the closely monitored anesthesia recovery phase, the animals were returned to their housing boxes. In the sham group, identical analgesic and anesthetic protocols were applied; however, these animals did not undergo thoracic trauma with HS and hind-limb ischemia (THS). Instrumentation was limited to incision and subsequent closure of the left thigh.

### Harvest and tissue preparation

Twenty-four hours after either THS or sham intervention, organ collection was performed under deep anesthesia with sevoflurane via exsanguination through cardiac puncture [20]. Following laparotomy, the jejunum was dissected from adjacent intestinal segments and a tissue section of approximately 3 cm in length was immersed in 3.7% formaldehyde for 24 h to achieve fixation. Upon rinsing, tissue dehydration was performed using an ethanol gradient prior to paraffin embedding. Tissue sections of 4 μm thickness were generated using a microtome for subsequent histological examination. Additional jejunal samples were flushed with ice-cold PBS and subsequently stored at −80 °C. Of note, a limited number of samples could not be re-analyzed because they had already been exhausted for pilot experiments to establish corresponding staining protocols and in a previously published study [20]. As a result, the sample size differed slightly between some histological analyses. However, this approach allowed alignment to the 3R principles and avoided the need for additional animal experiments.

### Hematoxylin and eosin (H&E) and periodic acid-schiff (PAS)-Alcian blue staining

Jejunal sections were deparaffinized, rehydrated, and stained with either hematoxylin and eosin (H&E) or PAS-Alcian blue. H&E staining involved a hematoxylin solution modified according to Gill III° (Sigma Life Sciences, St. Louis, USA) and a 1% eosin solution (Morphisto, Frankfurt am Main, Germany). PAS-Alcian blue staining included sequential incubations with Alcian blue (Sigma Life Sciences, St. Louis, USA), periodic acid, Schiff’s reagent, and a hematoxylin solution modified according to Gill III°. Sections were rinsed between each step. Subsequently stained sections were dehydrated and coverslipped using Neo-Mount™ mounting medium (Sigma Life Sciences, St. Louis, USA).

### Immunohistochemical stainings

For immunohistochemistry, deparaffinization and rehydration of jejunal tissue sections were performed prior to heat-induced antigen retrieval using EDTA buffer (pH 9.0; for CD3/ CDH1/ Ki-67) or citrate buffer (pH 6.0; for MPO). After cooling, tissues were permeabilized using 0.2% Triton X-100, and treated with Sudan Black B (Sigma-Aldrich, St. Louis, USA; for CD3/ MPO/ CDH1) or ReadyProbes™ Tissue Autofluorescence Quenching Kit (Thermo Scientific, Rockford, USA; for Ki-67) according to the manufacturer’s protocol to reduce autofluorescence. Blocking of non-specific binding was performed using antigen block solution (see Table 1). Primary antibodies were diluted 1:250 for CD3 (Anti-CD3 antibody [CD3-12] Rat monoclonal antibody, Abcam, Cambridge, UK), 1:200 for MPO (Human/Mouse Myeloperoxidase/MPO Antibody, R&D Systems, Minneapolis, USA), 1:100 for CDH1 (E-cadherin [24E10] Rabbit IgG monoclonal antibody, Cell Signaling Technology, Boston, USA), and 1:100 for Ki-67 (Rabbit Recombinant Monoclonal Ki67 antibody, Abcam, Cambridge, UK) and incubated overnight at 4 °C. Following rinsing, tissue sections were incubated with the corresponding secondary antibody in following dilutions: 1:300 with Goat anti-Rat IgG conjugated to Alexa Fluor™ 568 for CD3, 1:200 with Donkey anti-Goat IgG (H + L), Alexa Fluor™ 568 for MPO, 1:300 Alexa Fluor™ 488-conjugated Goat anti-Rabbit IgG for CDH1 and Ki-67 (all secondary antibodies from Thermo Scientific, Rockford, USA). Sections were mounted with Prolong Gold Antifade containing DAPI (Life Technologies, Eugene, USA). Fluorescence imaging was conducted using a Zeiss Axio Imager M1 microscope equipped with AxioVision SE64 Rel. 4.9 software (Zeiss, Oberkochen, Germany).

**Table 1:**
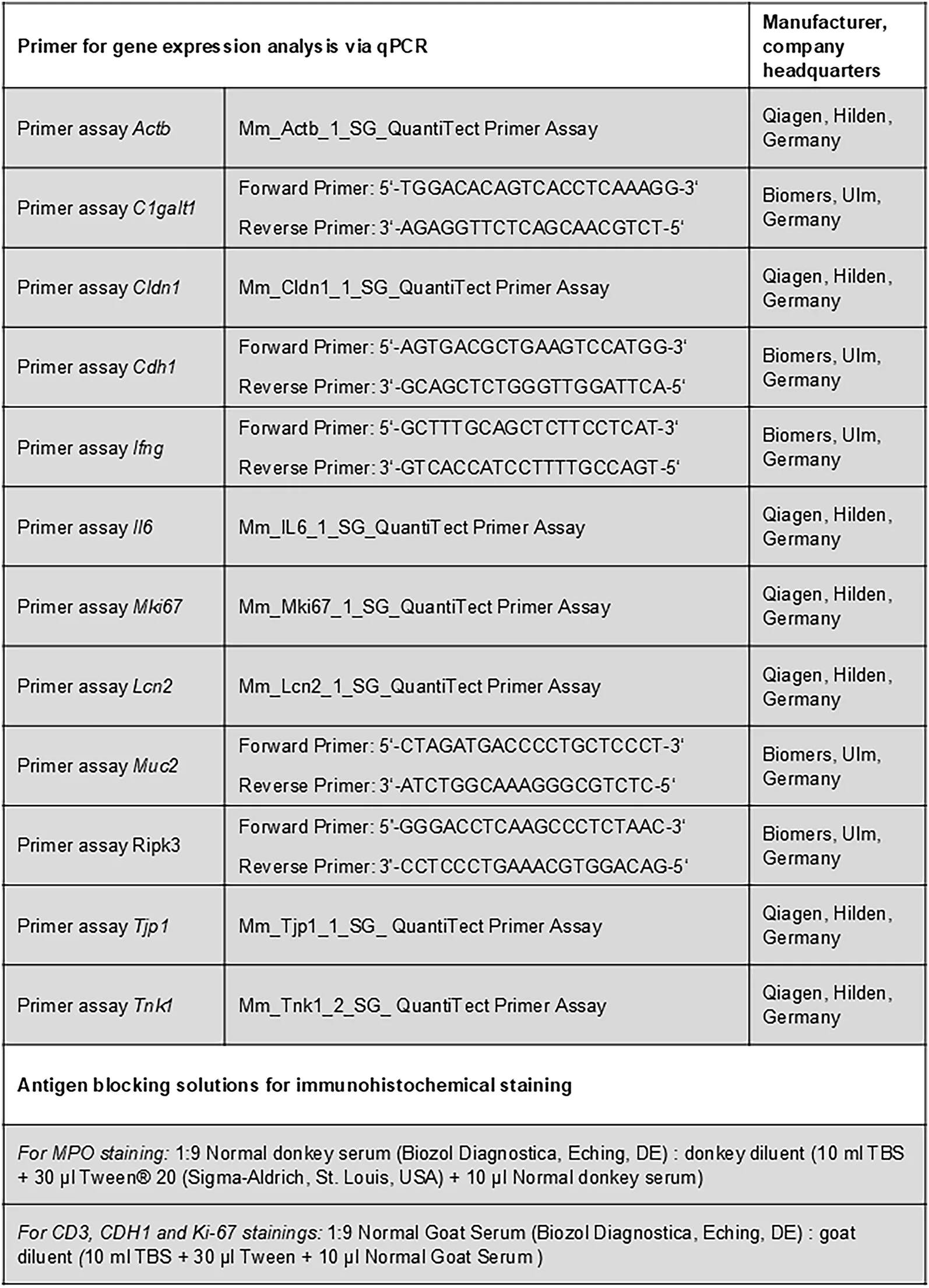
Listing of primers used for quantitative PCR (qPCR) alongside antigen blocking solutions applied for immunohistochemical staining of murine jejunal tissue

### Histological analysis

In H&E sections, villus morphology was primarily assessed in a descriptive manner by two independent observers blinded to the experimental groups, focusing on villus length, epithelial continuity, and presence or absence of edema, epithelial detachment, or necrosis. Quantification of goblet cells and mucus-covered regions was performed using QuPath-0.4.4 software [21]. Jejunal sections stained with PAS-Alcian blue were imaged at 20× magnification. To enable automated area recognition, six randomly chosen images were manually annotated to train the software in identifying mucus-positive regions. Ten villi per animal were selected for quantitative analysis.

### Quantitative polymerase chain reaction (qPCR)

Frozen jejunal tissue was placed in liquid nitrogen, preventing RNA degradation, and pulverized using an RNase-free (RNaseZAP™, Sigma-Aldrich) mortar and pestle. Samples were kept on ice until the beginning of RNA extraction using the RNeasy Plus Kit (Qiagen, Hilden, Germany), following the manufacturer’s instructions. RNA concentrations were measured using the Qubit™ RNA High Sensitivity Assay Kit (Qiagen, Hilden, Germany) according to the manufacturer’s protocol. For complementary DNA (cDNA) synthesis, 500 ng of RNA per sample was reverse-transcribed and diluted using the AffinityScript qPCR cDNA Synthesis Kit (Agilent, Santa Clara, USA) in accordance with the manufacturer’s protocol before storage at −20 °C. qPCR was performed in duplicate for determination of alterations in gene expression between THS-exposed and sham animals using the Brilliant III SYBR-Green qPCR Master Mix Kit on an AriaMx Real-Time PCR System (both from Agilent, Santa Clara, USA). Data were analyzed using AriaMx HRM qPCR software (Agilent, Santa Clara, USA). Primer sequences are listed in Table 1. Relative gene expression was assessed using the 2^−ΔΔCT method, with *Actb* as housekeeping gene.

### Statistical analysis

Statistical evaluation was performed using GraphPad Prism (version 10.0.0, GraphPad Software, Boston, USA). Data are displayed as individual values and as mean ± standard error of the mean (SEM). Given the limited sample size, normal distribution of the data was not assumed. Group comparisons were conducted using the non-parametric Mann-Whitney U test. Statistical significance was defined as *p* ≤ 0.05.

## Results

### Absence of remote injury to the jejunal epithelium in histological examination

**Fig. 1:**
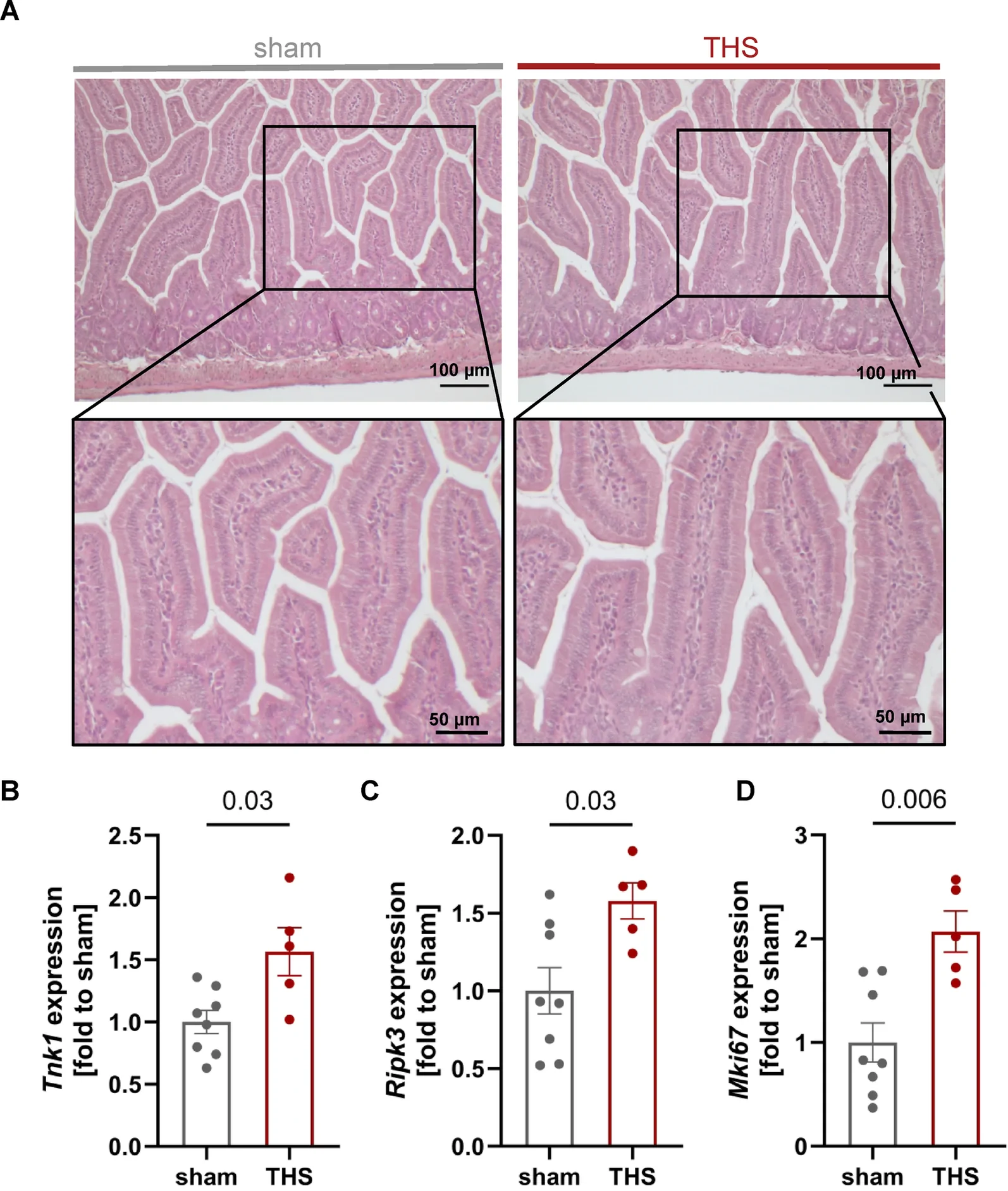
Histological and molecular assessment of epithelial damage and proliferation in the jejunum 24 h following blast-induced thoracic trauma with hemorrhagic shock and hind-limb ischemia (THS). (**A**) Representative hematoxylin and eosin (H&E)-stained jejunal sections from sham-treated and THS-exposed mice demonstrated intact epithelial architecture and preserved villus morphology, with no evidence of trauma-induced structural disruption. (**B**, **C**) However, cellular damage on molecular level was detected, as quantitative PCR analysis revealed significant upregulation of *Thirty-eight-negative kinase 1 (Tnk1)* and *Receptor-Interacting Serine/Threonine-Protein Kinase 3 (Ripk3)* in THS-exposed animals compared to shams. (**D**) Furthermore, *Ki-67 (Mki67)* gene expression was significantly increased, indicating enhanced proliferative activity. *n* = 5–8 per group. Statistical analysis was performed using the Mann-Whitney U test, with significance defined as *p* ≤ 0.05. Data are presented as mean ± standard error of the mean (SEM).

To evaluate the extent of intestinal tissue injury 24 h following experimental THS and to assess its potential impact on the structural integrity of the intestinal epithelium and villus morphology, an orienting histological evaluation of H&E-stained jejunal sections from sham- and THS-exposed mice was conducted (Fig. 1A). Microscopic analysis revealed no discernible histopathological differences between sham and THS and no indication of epithelial disruption was observed in both experimental groups.

### Increase of molecular indicators of epithelial injury and proliferation following THS

To further elucidate the impact of THS on jejunal epithelial integrity, we next analyzed gene expression profiles associated with epithelial damage and proliferative activity. Quantitative PCR analysis revealed a significant upregulation of apoptosis- and necroptosis-related markers, including *Thirty-eight-negative kinase 1 (Tnk1)* and *Receptor-Interacting Serine/Threonine-Protein Kinase 3 (Ripk3)*, indicating molecular evidence of remote epithelial injury in the jejunum of THS-exposed mice compared to sham-treated littermates (Fig. 1B, C). Notably, 24 h following THS, expression of the proliferation marker *Ki-67 (Mki67)* was also markedly increased (Fig. 1D), suggesting the onset of regenerative processes at the transcriptional level. However, this increase was not yet reflected at the protein level (Suppl. Fig. 1).

### Elevated expression of distinct barrier proteins in the jejunum after THS

**Fig. 2:**
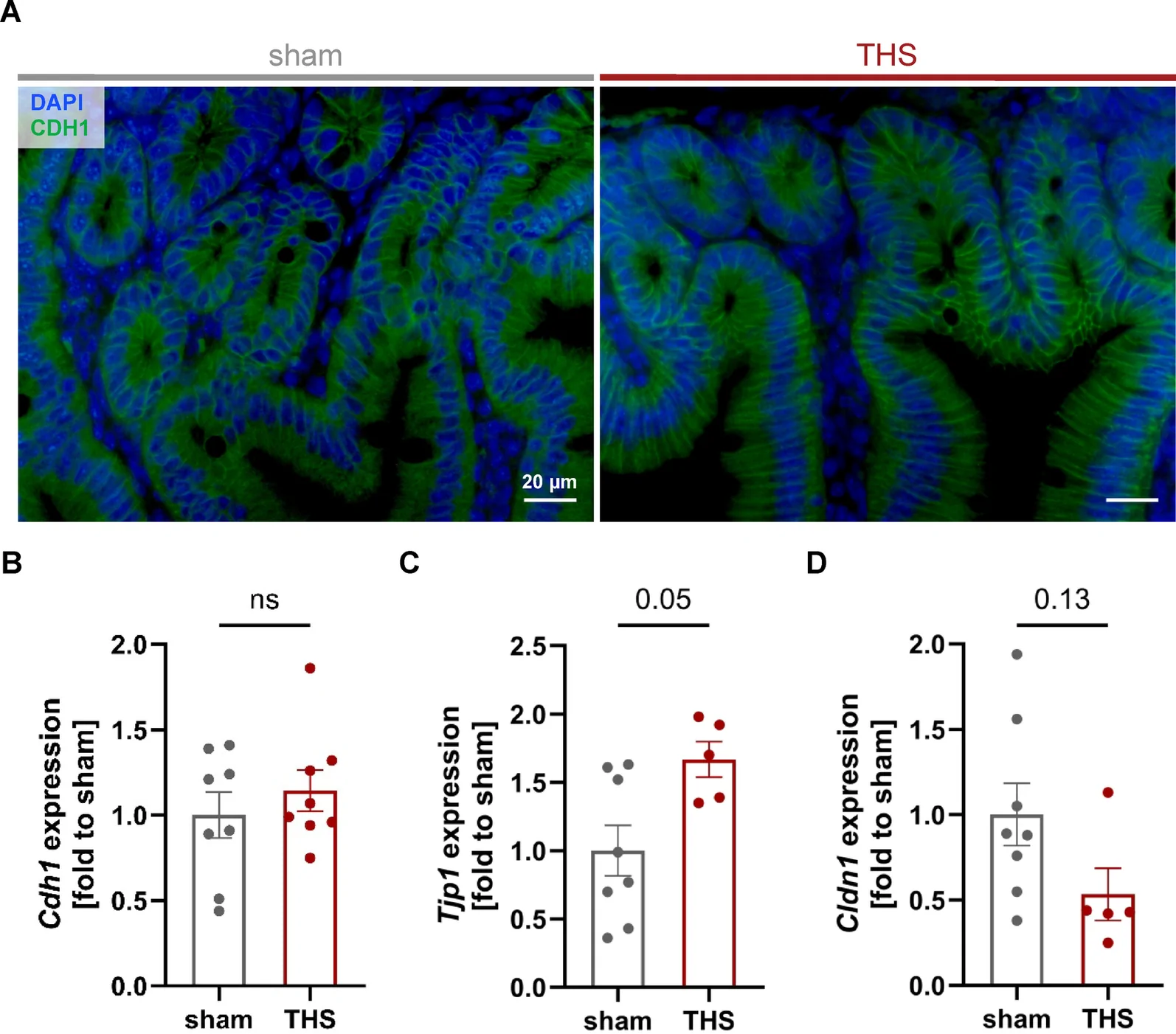
Analysis of junctional protein expression in jejunal tissue 24 h following blast-induced thoracic trauma with hemorrhagic shock and hind-limb ischemia (THS). (**A**, **B**) Protein and gene expression levels of the adherens junction protein E-Cadherin (CDH1) remained unaffected by THS. (**C**) In contrast, *tight junction protein 1/ zonula occludens 1* (*Tjp1/ ZO-1*) gene expression was significantly increased in THS-exposed mice compared to sham animals. (**D**) The expression of *claudin-1* (*Cldn1*) was not significantly altered in the THS-exposed group compared to sham mice; however, a trend towards a reduced expression can be noted. *n* = 5–8 per group. Statistical analysis was performed using the Mann-Whitney U test, with significance defined as *p* ≤ 0.05. ns, not significant. Data are presented as mean ± standard error of the mean (SEM).

The integrity of the intestinal barrier relies on a coordinated network of intercellular junctional proteins, including adherens and tight junctions, which regulate paracellular permeability and preserve epithelial cohesion [22]. To assess remote alterations in barrier integrity following THS, analysis of both gene expression and protein levels of key junctional components were conducted. Expression of the adherens junction protein E-cadherin (CDH1) remained unaltered at both gene and protein level (Fig. 2A, B). In contrast, *tight junction protein 1/ zonula occludens 1 (Tjp1/ ZO-1)* was significantly upregulated in THS-exposed mice compared to sham controls (Fig. 2C). Expression of *claudin-1 (Cldn1)* was not significantly affected by THS, yet, showing a non-significant decline (Fig. 2D).

### Assessment of jejunal mucus integrity following THS

**Fig. 3:**
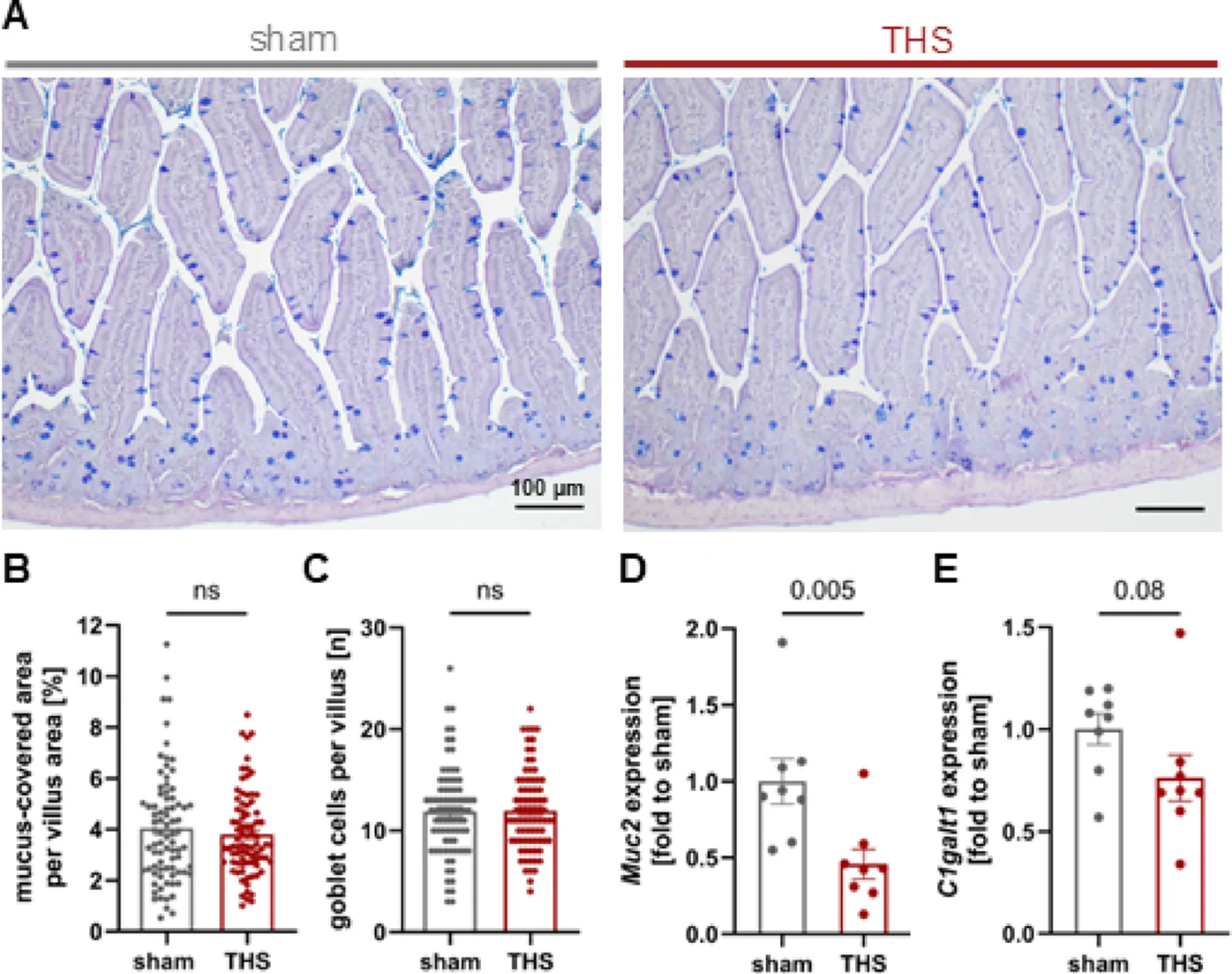
Effects of blast-induced thoracic trauma with hemorrhagic shock and hind-limb ischemia (THS) on jejunal mucus. (**A-C**) Histological evaluation of PAS-Alcian blue-stained sections showed no alterations in mucus-covered area or goblet cell counts between both groups. (**D**,** E**) Gene expression of key mucosal components, *mucin 2 (Muc2)* and *core 1 β1*,*3-galactosyltransferase (C1galt1)*, was assessed 24 h following THS or sham intervention, revealing a significant downregulation of *Muc2* in THS-exposed compared to sham mice. *C1galt1* showed a trend for reduced gene expression level in the THS group compared to sham counterparts; however, results did not reach statistical significance. B, C: *n* = 80; D, E: *n* = 8. Statistical analysis was performed using the Mann-Whitney U test, with significance defined as *p* ≤ 0.05. ns, not significant. Data are presented as mean ± standard error of the mean (SEM).

In addition to intercellular junctions, the mucus layer represents a critical component of the jejunal barrier, serving as first line of defense against luminal MAMPs and microbial translocation. Histological analysis of PAS-Alcian blue-stained jejunal sections was therefore performed to quantify the mucus-covered surface and assess goblet cell filling (Fig. 3A). The extent of mucus coverage remained comparable between sham-treated and THS-exposed animals (Fig. 3B), and goblet cell density did not differ between groups (Fig. 3C). Likewise, assessment of actively secreting goblet cells revealed no alterations in either absolute or relative numbers (Suppl. Fig. 2). To complement these histological findings, expression levels of key mucosal constituents, including *mucin 2 (Muc2)* and *core 1 β1*,*3-galactosyltransferase 1 (C1galt1)*, were analyzed. Despite the absence of histological changes, *Muc2* expression was significantly downregulated following THS, whereas *C1galt1* displayed a similar trend without reaching statistical significance (Fig 3D, E). These findings indicate that THS elicited detectable alterations in mucus-associated gene expression without affecting mucus secretion or goblet cell numbers at 24 h post THS.

### Remote alteration of inflammatory response in the jejunum following THS

**Fig. 4:**
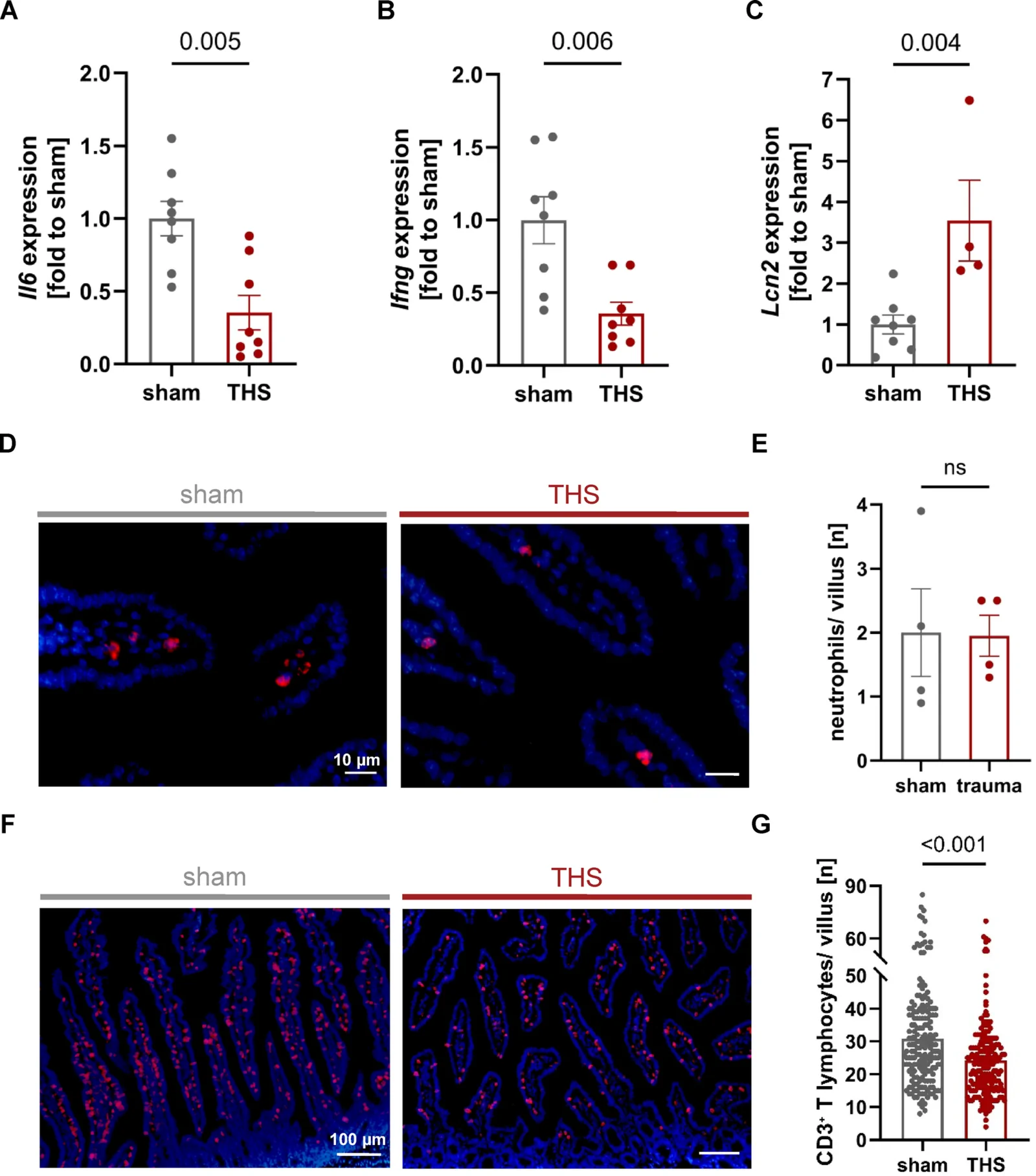
Post-traumatic inflammatory response in the jejunum following blast-induced thoracic trauma with hemorrhagic shock and hind-limb ischemia (THS). (**A**,** B**) Gene expression analysis 24 h post intervention revealed a significant downregulation of *interleukin-6* (*Il6*) and *interferon-γ* (*Ifng*) in THS-exposed mice compared to sham animals. (**C**) In contrast, *lipocalin 2 (Lcn2)* was significantly upregulated following THS in comparison to sham mice. (**D**,** E**) Immunohistochemical staining demonstrated an unaltered neutrophil infiltration, (**F**,** G**) while the number of CD3⁺ T lymphocytes in the jejunal mucosa was decreased in the THS group compared to shams. A–C: *n* = 4–8. E: mean values were derived from analysis of 30–45 villi per animal. F, G: *n* = 180. Statistical analysis was performed using the Mann-Whitney U test, with significance defined as *p* ≤ 0.05. ns, not significant. Data are presented as mean ± standard error of the mean (SEM).

Furthermore, the post-traumatic inflammatory response in the jejunal tissue was analyzed via gene expression profiling. *Interleukin-6 (Il6)* was significantly downregulated 24 h after THS compared to sham-treated mice (Fig. 4A). A similar reduction was noted for *interferon-γ (Ifng)* (Fig. 4B). In contrast, *lipocalin 2 (Lcn2 )* as an indicator for intestinal inflammation [23] was significantly upregulated following THS in comparison to sham counterparts (Fig. 4C). Neutrophil infiltration remained unchanged, as reflected by stable cell counts (Fig. 4D, E). In contrast, immunohistochemical analysis revealed a reduction in CD3⁺ T lymphocytes in the jejunal mucosa of trauma-exposed mice (Fig. 4F, G), which appears consistent with the reduced *Ifng* expression.

## Discussion

In the present post hoc analysis of a recently published murine model combining blast-induced thoracic trauma with transient hemorrhagic shock and hind-limb ischemia, we demonstrate, in addition to previously confirmed acute kidney injury [20], clear evidence of remote intestinal injury. Notably, classical signs of intestinal damage were absent on light microscopy, most likely due to the moderate severity of the thoracic trauma including HS. In contrast, pronounced intestinal morphological alterations are generally observed in models simulating more severe systemic injury patterns, such as polytrauma [19] or extended HS periods reaching a MAP of approximately 25 mmHg for up to 2 h [24], 2.5 h [6], or even 3 h [25] before resuscitation. In such severe settings, histological changes include villous stroma broadening, edema formation, focal necrosis, and epithelial cell detachment [19, 24].

However, evidence of increased intestinal *Mki67* expression 24 h post THS supports the notion that repair mechanisms were already progressing at the time of analysis. In a HS model, mucosal injury in both ileum and colon was largely resolved within 12 h, with barrier restoration completed by 24 h, suggesting that initial remote intestinal damage in the present moderate THS model may have already undergone repair [26]. This interpretation is further supported by the upregulation of the cellular proliferation marker Ki-67. Similarly, in a laparotomy trauma/HS rodent model, morphological villi damage peaked 1 h post injury and rapidly declined thereafter, while mucus thickness, as a functional correlate, was reduced within the first 3 h post trauma [27]. Apoptotic events, mainly localized at the crypt base, peaked within 3 h post polytrauma in rodents, correlating with trauma intensity [28]. As a major driver of intestinal apoptosis and multi-organ failure after combined polytrauma/HS, enhanced mRNA levels of *Tnk1* were identified within the small intestine as early as 4 h after injury [29]. In the present thorax trauma/HS/hind-limb ischemia experiment, intestinal *Tnk1* expression remained elevated even after 24 h, accompanied by increased levels of *Ripk3*, a molecular switch from apoptosis to necroptosis [30]. In agreement, kidney ischemia/reperfusion experiments demonstrated that intestinal RIPK3 overexpression at 24 h causes necroptosis and inflammasome activation, resulting in small intestinal damage via purinergic signaling [31].

Regarding posttraumatic alterations in intestinal barrier integrity, CDH1 gene and protein expression remained unchanged, whereas *ZO-1 (Tjp1)* expression significantly increased 24 h after THS. The discrepancy between gene and protein expression observed in our experiments may be attributable to the study limitation, that analyses were restricted to a relatively early single time point. Upon direct intestinal ischemia/reperfusion injury in rats, barrier permeability peaked within 1 h post injury, associated with pronounced tight junction disruption. However, the damaged intestinal villi were repaired within 6–24 h, accompanied by compensatory ZO-1 upregulation [32]. Similarly, in a controlled human model of 30 min of ischemia, barrier proteins, including E-cadherin and ZO-1, realigned within 120 min after reperfusion, reflecting remarkable intestinal repair capacity that may also apply to remote traumatic injury [33]. Mechanistically, hypoxic conditions, such as those accompanying thoracic trauma and HS, can induce hypoxia-dependent upregulation of epithelial barrier proteins [34]. This aligns with the pronounced splanchnic vasoconstriction and renal capillary perfusion deficits recently observed during the first 24 h post THS in the same model [20].

Yet, in a more severe rodent polytrauma/HS model, including direct intestinal and liver injury, tight junction disruption (e.g. occludin) has been reported up to 24 h post injury, leading to barrier dysfunction, which could be inhibited by early application of tranexamic acid [35]. Likewise, remote injuries, such as TBI in mice, led to a decrease in expression of intestinal ZO-1 and occludin by 6 h post injury [36]. Isolated sublethal HS reduced ZO-1 and claudin-3 protein levels in rat ileum at similar early time points [37]. Overall, it is tempting to speculate that a severe direct or remote trauma impact may lead to an early loss of intestinal tight junctions, which may, alongside potent repair mechanisms, rapidly return to normal expression profiles, whereas a moderate trauma may instead trigger compensatory upregulation of barrier proteins.

In the current thorax trauma/HS/hind-limb ischemia model, the mucus barrier, predominantly composed of mucin 2, produced by goblet cells [38], appeared modestly impaired. Whereas *C1galt1* expression, a key glycosyltransferase for mucin-type O-glycans [39], was only slightly reduced, and goblet cell counts remained stable, intestinal *Muc2* transcript levels were significantly reduced by approximately 50% at 24 h after remote THS. A previous study in human polytrauma [8] demonstrated early systemic appearance of MUC2 within the first hour after polytrauma, particularly under HS conditions, indicating that both direct and remote severe trauma compromise mucosal defense.

Regarding the inflammatory response in isolated murine thoracic trauma, pulmonary IL-6 expression peaks within 2 h but normalizes by 24 h [40]. In a similar combined THS model, systemic concentrations of IL-6 return to baseline approximately 20 h after the insult [41]. In the present study, however, the remote local response in the intestine revealed reduced *Il6* and *Ifng* expression levels, alongside lower T-cell counts, consistent with spatial compartmentalization of the immune response [42]. This redistribution may reflect preferential recruitment of immune cells, particularly T lymphocytes, to the primarily injured lungs, where the inflammatory molecular signature persists up to 24 h post trauma, and likely beyond [43].

The significant upregulation of intestinal *Lcn2* observed may partially reflect increased activity of neutrophils despite unchanged neutrophil counts; or by other local sources of lipocalin 2. Functionally, *Lcn2* is involved in intestinal inflammatory processes [23], and, as a protease-resistant protein, can limit bacterial growth by sequestering iron-containing siderophores, and exhibits anti-inflammatory features [44], suggesting some protective function upon intestinal upregulation.

Adaptive immune alterations were reflected by a modest but significant reduction in villous CD3⁺ T lymphocytes after extra-abdominal THS, suggesting compromised local defense. This observation supports the concept of host immune prioritization toward primary injury sites, at the expense of remote areas, to prevent systemic inflammation [45]. However, in another study at later time points, 48 h after murine femur fracture, CD3⁺ T lymphocytes were present in the gut-associated lymphoid tissue of the small intestine, but remained unaltered in the spleen 48 h after remote injury, supporting mucosal repair [46].

It is noteworthy, that based on the literature and present findings, there are currently no fully established, universally accepted biomarkers - either experimental or clinical - that comprehensively and quantitatively capture “intestinal injury” as a single entity. Our assessment therefore relies on a composite of molecular indicators (e.g. Tnk1, Ripk3, Muc2, Tjp1, Lcn2), barrier‑related changes, and immune cell alterations, which collectively suggest remote intestinal stress and adaptive barrier remodeling, but do not constitute a single validated injury score.

As limitations of the study, we employed a murine model combining blast-induced thoracic trauma, pressure‑controlled hemorrhagic shock, and hind-limb ischemia due to femoral vessel preparation and ligation. Accordingly, the intestinal alterations observed reflect the net effect of this composite extra‑abdominal insult and cannot be attributed to thoracic trauma alone. In the absence of thoracic‑trauma‑only and hemorrhagic‑shock‑only comparator groups, the specific contributions of lung injury, systemic hypotension, and limb ischemia to the remote intestinal changes, as well as potential lung-gut crosstalk, must be interpreted with caution.

Moreover, the sham group underwent anesthesia, analgesia, and thigh incision but not thoracic trauma, hemorrhagic shock, or hind-limb ischemia, so the comparison primarily contrasts combined thoracic‑trauma-shock-ischemia with isolated soft tissue trauma, rather than isolating the effect of shock per se. Our analyses were further restricted to a single time point (24 h), limiting insight into the temporal evolution of intestinal injury and repair. Finally, we relied on a panel of molecular, barrier‑related, and immunological readouts, in the context of the current lack of universally accepted biomarkers that comprehensively quantify intestinal injury, and we examined only male mice, which constrains extrapolation to female subjects and more heterogeneous populations.

In conclusion, using a standardized murine blast-induced thoracic trauma/HS/hind-limb ischemia mouse model, we demonstrate remote intestinal alterations, characterized by reduced mucin production, compensatory upregulation of distinct tight junction proteins, and decreased local T-cell abundance. Further experimental and clinical investigations should delineate the temporal and mechanistic features of the intestinal adaptive immune response, in relation to remote trauma severity and systemic hemodynamic compromise.

## Supplementary Information

Below is the link to the electronic supplementary material.

Supplementary Material 1.

Supplementary Material 2.

## Data Availability

Not applicable.

## Abbreviations

THS: Thoracic trauma with transient hemorrhagic shock and hind-limb ischemia
DAMPs: Damage-associated molecular patterns
MAMPs: Microbe-associated molecular patterns
MODS: Multiple organ dysfunction syndrome
HS: Hemorrhagic shock
HMGB1: High-mobility group box 1
GBB: Gut-blood barrier
TBI: Traumatic brain injury
ZO-1: Zonula occludens 1
MAP: Mean arterial pressure
H&E: Hematoxylin and Eosin
PAS: Periodic Acid-Schiff
CDH1: E-cadherin
cDNA: Complementary DNA
SEM: Standard error of the mean
TNK1: Thirty-eight-negative kinase 1
RIPK3: Receptor-Interacting Serine/Threonine-Protein Kinase
TJP1: Tight junction protein 1
CLDN1: Claudin-1
MUC2: Mucin 2
C1galt1: Core 1 β1,3-galactosyltransferase 1
IL6: Interleukin-6
IFNG: Interferon-γ
Lcn2: Lipocalin 2
